# Exploring physiological and molecular dynamics of drought stress responses in plants: challenges and future directions

**DOI:** 10.3389/fpls.2025.1565635

**Published:** 2025-03-24

**Authors:** Sajad Ali, Rakeeb Ahmad Mir, Md Azizul Haque, Danishuddin, Mohammed A. Almalki, Mohammad Alfredan, Ashraf Khalifa, Henda Mahmoudi, Mohammad Shahid, Anshika Tyagi, Zahoor Ahmad Mir

**Affiliations:** ^1^ Department of Biological Sciences, College of Science, King Faisal University, Al-Ahsa, Saudi Arabia; ^2^ Department of Biotechnology, School of Life Sciences, Central University of Kashmir, Ganderbal, India; ^3^ Department of Biotechnology, Yeungnam University, Gyeongsan, Republic of Korea; ^4^ Directorate of Programs, International Center for Biosaline Agriculture, Dubai, United Arab Emirates; ^5^ Department of Agricultural Microbiology, Faculty of Agriculture Sciences, Aligarh Muslim University, Aligarh, India; ^6^ Department of Plant Science, University of Manitoba, Winnipeg, MB, Canada

**Keywords:** drought, signaling, calcium, drought memory, ABA, soil properties, microbiota

## Abstract

Plants face multifactorial environmental stressors mainly due to global warming and climate change which affect their growth, metabolism, and productivity. Among them, is drought stress which alters intracellular water relations, photosynthesis, ion homeostasis and elevates reactive oxygen species which eventually reduce their growth and yields. In addition, drought alters soil physicochemical properties and beneficial microbiota which are critical for plant survival. Recent reports have shown that climate change is increasing the occurrence and intensity of drought in many regions of the world, which has become a primary concern in crop productivity, ecophysiology and food security. To develop ideas and strategies for protecting plants against the harmful effects of drought stress and meeting the future food demand under climatic calamities an in-depth understanding of molecular regulatory pathways governing plant stress responses is imperative. In parallel, more research is needed to understand how drought changes the features of soil, particularly microbiomes, as microorganisms can withstand drought stress faster than plants, which could assist them to recover. In this review we first discuss the effect of drought stress on plants, soil physicochemical properties and microbiomes. How drought stress affects plant microbe interactions and other microbe-driven beneficial traits was also highlighted. Next, we focused on how plants sense drought and undergo biochemical reprogramming from root to shoot to regulate diverse adaptive traits. For instance, the role of calcium (Ca^2+^), reactive oxygen species (ROS) and abscisic acid (ABA) in modulating different cellular responses like stomata functioning, osmotic adjustment, and other adaptive traits. We also provide an update on the role of different hormones in drought signaling and their crosstalk which allows plants to fine tune their responses during drought stress. Further, we discussed how recurrent drought exposure leads to the development of short-term memory in plants that allows them to survive future drought stresses. Lastly, we discussed the application of omics and biotechnological-based mitigating approaches to combat drought stress in sustainable agriculture. This review offers a deeper understanding of multiple factors that are related to drought stress in plants which can be useful for drought improvement programs.

## Introduction

Environmental stressors like drought severely damage plant growth and productivity across the globe (Koua et al., 2021; Khan et al., 2024). Global warming increases temperatures which cause evaporation, reduces surface water and dries out soils quickly all of which leads to drought stress (Passioura and Angus, 2010; Devincentis, 2020). Furthermore, drought stress is becoming more often and more intense due to climate change and global warming, which will have a direct impact on ecosystems and agriculture (Seleiman et al., 2021). The loss of arable agriculture land due to drought stresses further threatens food security, hence, necessitates a continued increase in yield by developing stress tolerant and high yielding crops. Between 2005 and 2015, an estimated USD 29 billion economic losses were associated with the drought (Trenberth et al., 2014; FAO, 2021). According to the FAO (2021), the current drought has a significant influence on global crop output and will continue to create yield changes from year to year. By 2050, 50% of arable land is expected to be under drought stress. In the past, many studies have revealed the impact of drought on crop yield losses in different regions across the world. For instance, in united states prolonged drought stress causes 21% yield losses in maize crops which leads huge economic losses (Boyer et al., 2013). According to Beillouin et al., 2020, drought stress causes 40% yield losses in wheat and barley crops which emphasizes the devastating effects of drought on agriculture. From 2006-2007, it was reported that drought stress leads huge yield losses in cotton (50%), barley (56%) and wheat (58%) grown in Australia (Roy et al., 2021). These case studies indicate the threat of drought stress on sustainable agriculture which can be furthermore threatening due to the rise of climate change and global warming. According to IPCC 2021, many regions in the world have already been severely affected by drought and are likely to prone future droughts because of the climate change which could increase hunger rate and threat to global food security. Therefore, there is need to find long term solutions to tackle future drought threat in sustainable agriculture which should implement a joint strategy, involving scientists from different fields with diverse expertise and resources.

Drought hinders natural plant development patterns, interferes with water relations and lower water usage efficiency. Drought stress also alters plant physiological traits such as stomatal functioning, photosynthesis, gaseous exchange, antioxidant system, transpiration rate all of which leads to growth retardation (Raza et al., 2023). Additionally, drought stress affects plants cell wall and cell membrane integrity which leads to cell death. Plants under drought stress shows high levels of oxidative stress that also affects plants growth and development. Plants under drought stress show different symptoms such as leaf rolling, wilting, stunning plants, early senescence, and leaf scorching (Corso et al., 2020). It has been calculated that severe droughts diminish carbon (C) absorption by 0.14 PgC yr^−1^ worldwide and inflict yearly losses of almost 1% of Earth’s terrestrial ecosystem function (Du et al., 2018). Furthermore, although sufficient moisture exists in the root zone, there are situations in which plants are unable to absorb water from the soil, a condition known as pseudo-drought or physiological drought (Daryanto et al., 2020). On the other hand, drought also alters plants’ response to other abiotic and biotic stressors which will further impact crop growth and yield (Fichman and Mittler, 2020). For instance, Vile et al. (2012) examined how Arabidopsis growth attributes were affected by drought and heat stress, and their combination. Both pressures dramatically decreased plant growth, and their combined effects were substantially more harmful. Many studies have revealed the effect of drought along with other abiotic stress combinations like heat, heavy metals, cold and salinity which has significant detrimental effect on plant growth as compared to drought alone (Rizhsky et al., 2004; Vile et al., 2012; Sales et al., 2013). Similarly, drought stress also negatively regulates plant immune response to pathogens (Ali et al., 2023a). For example, many studies have reported that drought stress can enhance the disease severity of some of the important diseases such as charcoal stalk rot in sorghum (Ramegowda, et al., 2015), smut in cereals (*Urocystis agropyri*, Colhoun, 1973), and root rot in safflower (Phytophthora sp., Duniway, 1977). These studies further highlight the impact of drought in plant responses to other stressors therefore future studies are required to understand the crosstalk between drought and other stressors and their influence on growth and plant defense, which will pave the way for developing future multifactorial resistant crops. Therefore, understanding the molecular underpinnings of drought response in plants and identifying key players involved in drought tolerance are crucial for crop breeding and genetic engineering programs to develop resilient crops.

## Effect of drought stress on soil properties and microbiome

Soil physicochemical properties, moisture content, microbiome, and nutritional content are important indicators of plant health (Pautasso et al., 2018; Gupta et al., 2020). Any change in the above indicators has a direct impact on plant development. Climate change-driven environmental stressors like drought and inadequate management practices are expected to increase the frequency and severity of disturbances that alter the microbiome structure, nutritional composition of soil or deplete water resources, and soil physicochemical properties, (structure, porosity, and pH), which can directly affect crop output. In addition, drought also causes a reduction in plant carbon inputs because of early senescence in dry conditions (Schaeffer et al., 2017). Like how it affects soil carbon dynamics, drought also affects nitrogen dynamics directly and indirectly (Hartmann et al., 2015), as water availability is crucial for microbial processes fixing and transforming nitrogen, thereby lowering soil nitrogen cycle rates (Homyak et al., 2017). Drought significantly decreased soil respiration, microbial biomass, carbon, and nitrogen which also affect plant growth (Liu et al., 2023).

Plant roots nourish complex microbial communities which have a significant influence on plant growth, nutrition, and overall health (Ali et al., 2023a, 2023b). Although the composition and geographic compartmentalization of these communities has been thoroughly characterized in a variety of plant species, the effects of abiotic stressors on the root microbiota are still poorly understood (Ali et al., 2022). Robust analyses of microbiomes associated with roots in several plant systems have provided a significant understanding of the elements influencing the formation of communities. To prevent drying out, roots can alter their structure (Pautasso et al., 2018) and use resources (Santos-Medellín et al., 2021). Under stress, root exudation patterns can also change which may have an impact on rhizospheric characteristics (Song et al., 2018). In plants drought stress alters plant metabolism and root exudates chemistry which in turn influences the root microbiome and its structure. A key factor in the selective recruitment of rhizosphere microbial communities is the presence of plant exudates, which include sugars, amino acids, fatty acids, organic acids, vitamins, carboxylic acids, flavonoids, benzoxazinoids, and ethylene (ET) (Vives-Peris et al., 2020). Previous studies have reported that drought stress promotes the synthesis of glycerol-3-phosphate (G3P) that shapes Actinobacteria members in the rhizosphere, which enhances plant health and fitness under water deficit conditions (Xu et al., 2018). According to Lebeis et al. (2015), a decrease in salicylic acid (SA) production during drought stress also has a substantial effect on the development of the endogen and exo-microbiomes. Drought decreases the amount of iron (Fe) and phyto-siderophores available in the rhizosphere, which benefits actinobacteria, to flourish in such conditions and enhance plant performance (Xu et al., 2021). Under drought stress, several plant species diminished diderm bacteria in the roots and rhizosphere and attract monoderm bacteria, which are resistant to dryness because they have thicker cell walls thus could improve plant growth and stress tolerance (Naylor et al., 2017). The production of polysaccharides that enhance soil structure and water-holding capacity, the synthesis of indoleacetic acid (IAA), deaminase, and proline (Pro), and enhanced water circulation through fungal mycelia are some of the major ways that beneficial microbiota helps plants adapt to drought stress (Milošević et al., 2012). In poplar drought stress causes major shift in microbiome structure with more dominant phyla such as Actinobacteria, Firmicutes, and Proteobacteria (Xie et al., 2021). This study also identified subgroups of microbes, such as *Bacillus arbutinivorans*, *B. megaterium*, *B. endophyticus*, *Streptomyces rochei*, *Trichoderma ghanense*, *Penicillium raperi*, *Aspergillus terreus*, *Gongronella butleri*, and *Rhizopus stolonifer*, in drought-treated poplar rhizosphere soils. These studies further support the notion that drought stress alters the microbiome structure in plants and soil, however, it remains largely unknown on how drought influences unique microbiomes. Therefore, further investigation is needed to underpin the molecular mechanism of plant mediated response to shape unique microbiome during drought stress. Furthermore, it is essential to investigate at the molecular level how specific microbiomes are able to resist and thrive under drought conditions, with the goal of understanding their drought tolerance and host selection mechanisms. Future studies should also focus on examining the influence of plant genotype and soil properties on microbiome assembly during drought stress which can open new directions in harnessing the stress resilient microbiome in sustainable agriculture. Owing to their ability to flourish in extreme environments, microbiome driven drought tolerance in plants is the most viable strategy for improving crop production.

**Figure 1:**
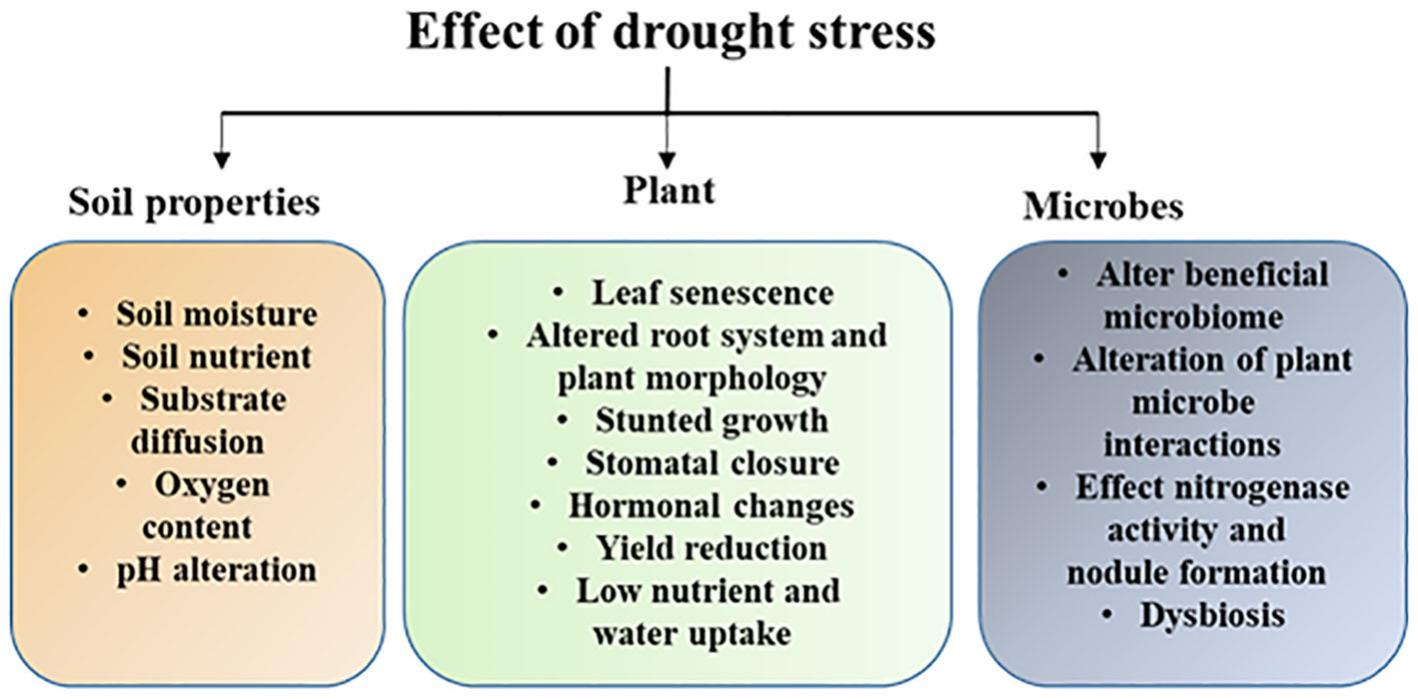
Schematic illustration shows the impact of drought on plants, soil physicochemical properties and soil microbiome. Drought alters plant morphological and biochemical properties that leads growth inhibition. In soil drought reduces soil moisture which has huge impact on soil properties and microbiome structure and their functional traits.

Drought can also have a detrimental effect on soil microorganisms and their functional attributes like inhibiting enzyme activity, nitrogen fixation, and disrupting nutrient cycling (such as C, N, and P), which can affect plant growth and adaptive responses (He et al., 2014; Nong et al., 2023). Beneficial plant-microbe relationships may be disrupted by changes in the global environment, such as agricultural practices and climate change. For example, drought has the potential to cause a variety of intricate changes in microbial dynamics, including both positive and negative effects on mycorrhizal associations’ essential players influencing plant performance (Xu et al., 2018). Future studies are required to unravel the complex relationships that exist between plants and their microbiome during drought stress in different crop systems at the ecological, molecular, and evolutionary levels which will enhance our understanding and help to develop microbial-based strategies to increase host fitness and drought resistance. The impact of drought stress on plants, soil physicochemical properties, and microbes is shown in **Figure 1**.

## Plant responses to drought stress

During drought stress, plants face serious challenges due to water scarcity. High osmolality or drought stress causes dehydration, which reduces turgidity and pressure against the cell wall—two factors necessary for preserving plant development and structure (Raza et al., 2013; Ali et al., 2022). Other challenges plants face during drought stress is energy crisis due to photosynthesis inhibition and production of harmful metabolites. However, to survive under drought, plants exhibit several behaviors such as taking up water from their extensive and deep root systems, growing smaller, more succulent leaves, increasing their diffusive resistance, decreasing the rate at which water is lost through leaf wilting, and slowing down transpiration (Farooq et al., 2012). Numerous changes in phenotypic architecture are observed in plants, including the formation of vascular bundle sheaths and thicker cuticles on the epidermis, smaller and denser stomata, a larger ratio of palisade to spongy parenchyma thickness, and epidermal trichomes (Silva et al., 2018). To counteract the water scarcity, stressed plants maintain their osmotic adjustment by increasing the amount of sugar in their leaves and roots (Miranda et al., 2020). Furthermore, during drought exposure, plants undergo morphological changes (phenotypic plasticity) (Basu et al., 2016), and physiological changes including cell membrane stability and osmotic adjustment (Abid et al., 2018). At biochemical level, plants produce a wide range of signaling molecules such as calcium, ROS, and hormones like ABA which reduces water loss via stomatal closure and triggers defensive mechanisms against drought (Kim et al., 2024). Among them, ABA is a major player which modulates distinct protective responses in plants during drought stress such as encoding LEA proteins, osmoprotectant biosynthesis, chaperones, aquaporins, sugar and proline transporters, and detoxification enzymes to eliminate ROS (Yamaguchi-Shinozaki and Shinozaki, 2006; Finkelstein, 2013; Yoshida and Fernie 2024). Further we have elaborated how plants sense drought signals and undergo biochemical reprograming which modulate plant response to water scarcity in below sections.

## Drought perception in plants

Plants can detect water deficit conditions in the soil and transmit the drought signals from the below-ground organs (roots) to the above-ground parts (leaves) to acclimatize to drought via ABA accumulation (Nong et al., 2023). Many players such as calcium channels, RBOHs, pH, hormones, peptides, and other secondary signaling molecules are linked with long-distance drought signaling from root to shoots. Among them, ABA is known to be crucial for driving major drought systemic signaling in plants. Discovery of ABA receptors and deciphering the fundamental ABA-signaling route has been one of the most significant developments in drought signaling in plants. For instance, the identification of PYR/PYL/RCAR also known as PYL as ABA receptors was one of the key findings in stress signaling in plants that open new frontiers in drought signaling (Kuromori et al., 2022). Also, the identification of PP2C and SnRK2 further unwires the complexity of ABA-mediated drought signaling response in plants (Kuromori et al., 2022). Plants detect water deficiency through the stomata and trigger intracellular and organ systemic signaling. ABA-induced stomatal closure and decreased transpiration water loss during drought stress are mediated by plasma-membrane proteins, such as the anion channel SLAC1, which are SnRK2 substrates (Ma et al., 2009). However, plants also respond to drought stress through an ABA-independent manner (Geiger et al., 2009). Furthermore, unique alterations in gene expression, physiology, and metabolism are observed in plants under drought stress, providing additional evidence that plants possess an advanced sensing system for identifying drought stress, which triggers a cascade of adjustments at different cellular compartments. Numerous investigations have revealed that the responses to drought stress are mediated by electric and hydraulic signals, calcium waves, ROS, and phytohormone movements (Takahashi et al., 2020). Calcium channels such as REDUCED HYPEROSMOLALITY INDUCED Ca^2+^ INCREASE 1 (OSCA1), OSCA1.2/CALCIUM-PERMEABLE STRESS-GATED CATION CHANNEL 1 (CSC1) (Kudla et al., 2018), and MCA1 is a homolog of yeast MID1, a Ca^2+^permeable stretch-activated channel component are potential osmosensors that are activate during drought stress (Liu et al., 2018). The drought-induced activation of these calcium channels regulates different traits such as stomatal functioning. In addition to calcium, drought-induced systemic signaling is driven by ROS wave which is produced by respiratory burst oxidase D (RBOHD) and regulates stomatal closure (Yoshimura et al., 2021). Recently discovered H_2_O_2_ sensor HYDROGEN-PEROXIDE-INDUCED Ca^2+^ INCREASE (HPCA) in guard cells provide novel insights on the role of ROS in stress signaling. During stomatal closure, HPCA1, a leucine-rich repeat receptor-like kinase (LRR-RLK), causes Ca^2+^ influx into guard cells by activating its extracellular domain in response to H_2_O_2_ (Zandalinas et al., 2020). Future research should examine the function of cell wall sensors, ROS, calcium channels in drought signaling and how these factors affect the morphological, anatomical, biochemical, and physiological adaptations of plants, especially in the roots. Drought-induced reductions in water potential (ψw) and water constraint produce a variety of alterations in plant growth and development. A few of these modifications, including stomatal closure to regulate water loss from leaves, enable the plant to store water and prevent low ψw. Other adaptations, like solute accumulation and osmotic adjustment, help the plant withstand low ψw by preserving water and turgor or lessening the harmful effects of tissue dehydration (Ma et al., 2009). On the other hand, osmosensing in plants also entails detecting the effects of osmotic stress during drought stress on cellular elements like the cell wall and plasma membrane (mechanical stress brought on by plasmolysis, depolarization of the plasma membrane, and damage to the cell wall and plasma membrane) (Takahashi et al., 2020; Liu et al., 2018). How plants sense water potential (ψw), and osmotic stress is not fully understood, however, many players such as AtHK1, calcium channels and RLKs are known to be the possible sensing players in plants which warrants further investigation. For instance, ATHK1/AHK1 has been identified as one of the osmotic stress sensor because loss or gain of its function proved that it modulates drought response in Arabidopsis *via* ABA accumulation and induced expression of stress resistant genes (Tran et al., 2007; Wohlbach et al., 2008). Similarly, RPK1/BAK1 type of LRR-RLK has been reported to positively modulate ABA-induced stomatal closure in plants during drought stress (Collin et al., 2021). Nonetheless, significant knowledge gaps persist regarding apoplast signaling and its relationship with cytosolic signaling pathways during drought stress, indicating a need for further investigation.

**Figure 2:**
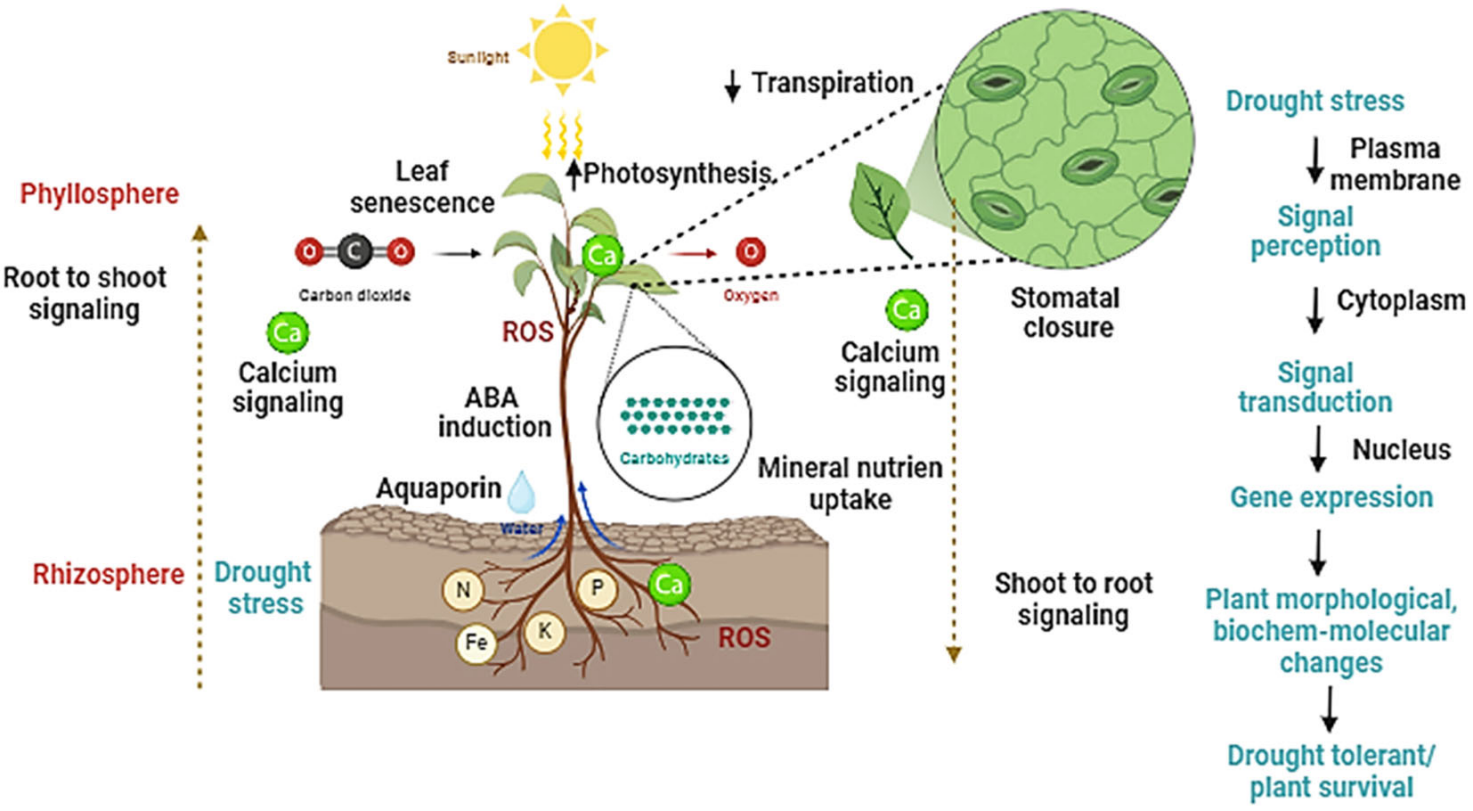
A schematic representation showing how plants perceive drought stress and undergo rapid physiological and biochemical reprograming at different cellular levels. The role of calcium, ROS, ABA and sugars in facilitating long-distance drought communication from roots to aerial parts, which regulates several functional features of plants such as photosynthesis, stomatal function, leaf senescence, and absorption of water and nutrients are highlighted in the figure.

A class of protein kinase namely sucrose nonfermenting 1 (SNF1)-related protein kinase 2s (SnRK2s) has been identified in *Arabidopsis thaliana* as an important signal transmitter of drought stress (Lin et al., 2020). Previous studies have shown that dehydrated leaf vasculature has elevated expression of the NINE CIS EPOXYCAROTENOID DIOXYGENASE3 (NCED3) gene, which codes for a crucial enzyme in the production of ABA which further supports that ABA is a key regulator for drought signaling in plants. It was recently demonstrated that the CLAVATA3/EMBRYO-SURROUNDING REGION-RELATED25 (CLE25) peptide serves as a long-distance signal for the synthesis of ABA in leaves that is generated from the roots (Soma et al., 2020). Drought also triggers systemic response’s, leading to systemic acquired acclimation (SAA), wherein localize tissues triggers responses in distant tissues. In addition to long-range electrical and hydraulic signals, ROS waves and calcium are important components of systemic acquired acclimation during drought (Yoshida and Fernie, 2018). Roots detect hydraulic signals, which are early indicators of drought stress in plants. It is unknown, yet how hydraulic signals are detected by the roots. Roots show distinct response during drought stresses such long and deeper root system, hydrotropic response. Many genes such as MIZU-KUSSEI1 (*MIZ1*) (Miller et al., 2009), involved in hydrotropic response, DEEPER ROOTING1 (*DRO1*) (Dietrich et al., 2024), promoting roots to grow downwards to escape drought stress. However, there remain many knowledge gaps how roots sense drought signals and undergo morphological and anatomical changes during drought stress warrants future investigation. Further we have shown how drought stress is perceived by plants that leads to series of changes at molecular, biochemical and physiological levels which regulate different growth and adaptive responses **Figure 2**.

## Hormonal interplay during drought stress

During drought stress, plants under go hormonal reprograming which have essential roles in growth, development, and adaptive responses or balancing growth and stress tradeoffs. Plants show many physiological, biochemical and morphological changes to cope with drought stress which are mainly regulated by hormones and their cross talk. Therefore, hormonal coordination is critical for plant responses to drought stress. Plant hormones like ABA, jasmonic acid (JA), salicylic acid (SA), ethylene (ET), auxin, brassinosteroid (BR), gibberellic acid (GA), and cytokinin (CK), are known to regulate distinct growth and adaptive responses during environmental stressors (Uga et al., 2013). However, their role in drought is not dully understood except ABA which is the major hormone that participates and regulates diverse host responses during drought stress. The signal perception and transduction during drought stress is regulated by ABA dependent and ABA independent pathway (Muhammad Aslam et al., 2022). Both pathways further activate the transcription factors such as, *MTC, bZIP, DREB* and *NAC* which lead to the expression of drought responsive elements. Similarly, the role of JA responsive genes in drought stress is also validated in different crop plants. Drought stress activates the JA precursor molecule, 12-OPDA, resulting in an increase in JAZ1 protein and activating the DREB gene to offer tolerance against drought stress (Wang et al., 2021). Several drought stress responsive genes have been found and characterized in crops because of significant advancements achieved in contemporary genetics and functional genomics techniques like transcriptomics, proteomics, and metabolomics. These key genes mainly code for proteins that have either metabolic or regulatory roles, such as those involved in detoxification, osmolyte biosynthesis, proteolysis of cellular substrates, water channel, ion transporter, heat shock protein (HSP), and late embryogenesis abundant (LEA) protein (Joshi et al., 2016). On the other hand, the regulatory class primarily comprises of TFs (*AREB, AP2/ERF, NAC, bZIP, MYC*, and *MYB*), signaling protein kinases mitogen activated protein kinases (MAPK), calcium-dependent protein kinases (CDPK), receptor protein kinases and protein phosphatases (phosphoesterases and phospholipase), which triggers signal transduction and expression of genes during stress responses (Wani et al., 2013).

ABA is one of the primary plant hormone that controls a variety of biological processes in plants during drought (Muhammad Aslam et al., 2022). For instance, drought induced ABA can reduce transpiration water loss and gas exchange by triggering stomatal closure. Additionally, ABA promotes root cell elongation and gradually raises hydraulic conductivity, allowing plants to recover from water-limited circumstances (Wani et al., 2013; Yu et al., 2017). ABA mediates drought stress by two pathways, ABA dependent and ABA independent. The ABA dependent pathway relies on the ABRE element, which plays an important role in ABA signaling and leads to the activation of downstream processing genes. Similarly, the role of ABA signaling components is reported to be involved in drought response mechanisms in woody plants (Yu et al., 2017). Arabidopsis NINE-CISEPOXYCAROTENOID DIOXYGENASE 3 (NCED3)mutants showed alterations in transcriptome and metabolome during drought stress which further highlights the important role of ABA in drought stress (Urano et al., 2009). Following exogenous ABA treatment, the genome of *Populus tremuloides* exhibited an upregulation of *AREB/ABF* elements [54]. Under drought conditions, transgenic plants overexpressing *AREB3* displayed a robust drought resistance phenotype with reduced biomass output (Yu et al., 2017). However, TFs like DREBs and NACs control the ABA-independent pathway, which is crucial in regulating drought stress. Dehydration-responsive element DRE (A/GCCGAC) is mainly dominant in drought-responsive elements. The ABA-independent activation of drought-responsive genes is facilitated by *DREB2A*, which is primarily activated by dehydration and marginally stimulated by ABA (Kim et al., 2011). Owing to its critical function in drought signaling researchers have established model for ABA perception and signal transduction in plants which is mainly controlled by three players such as PYR1) and PYR1-LIKE (PYR1/PYL ABA receptors, SnRK2 or SRK2 as signaling inducer and negative regulator or suppressor PP2C respectively (Cutler et al., 2010; Finkelstein, 2013).

Jasmonic acid is an important plant defense hormone that regulates different plant responses during biotic and abiotic stressors (Ali et el., 2017) For instance, JA triggers accumulation of defensive proteins, cell wall modification, stomata regulation and different plant developmental regulations. Many studies have shown that drought stress leads to the accumulation of JA which could modulate different responses. For instance, JA signaling Arabidopsis mutants such as *coi1-2*, *jaz1*, and *myc2-2* show drought sensitivity, demonstrating the importance of JA signal transduction mediated responses during drought (Song et al., 2022). On the other hand, exogenous treatment JA mitigated the negative effects of drought-induced membrane damage in barley (Bandurska et al., 2003). Also, overexpression of genes involved in JA signaling, biosynthesis, and JA-mediated stress responses provides drought tolerance in different plants (Liu et al., 2016). For instance, overexpression of allene oxide synthase (AOS) in potato plants provides drought tolerance by driving the induction of drought tolerant genes (Harms et al., 1995). Expression of *OsJAZ1-* gene in Rice showed improved resistance towards drought stress and its mutants were sensitive and hence suggested its role in mediating drought stress (Allagulova et al., 2022). Similarly, exogenous application of MeJA increases resistance towards drought stress by mediating the expression of proline and maintaining the antioxidant activity in wheat seedlings (Seo et al., 2011). Further the basic helix–loop–helix protein *OsbHLH148*, acts as transcriptional regulator of *OsDREB1* and *OsJAZ*, that are involved in signaling during drought stress (Ge et al., 2016). In drought tolerant *Prunus armeniaca* genotype, accumulation of JA promotes leaf senescence, reduces water loss and increased plant survival under drought conditions (Fleta-Soriano and Munné-Bosch, 2016). These studies further support the notion that JA plays a key role in drought response in plants. However, it remains largely unknow whether JA directly regulates drought response or involve other hormonal interplay like ABA which needs future attention. Also, in future it will be interesting to explore how JA signaling regulates drought responses in the presence of other stressors and its cross talk with other stress hormones and their impact on plant growth and stress responses. How accumulation of ABA mediates the activation of JAs is still elusive and the balance of these hormones during drought stress is crucial for development of drought tolerant cultivars.

**Figure 3:**
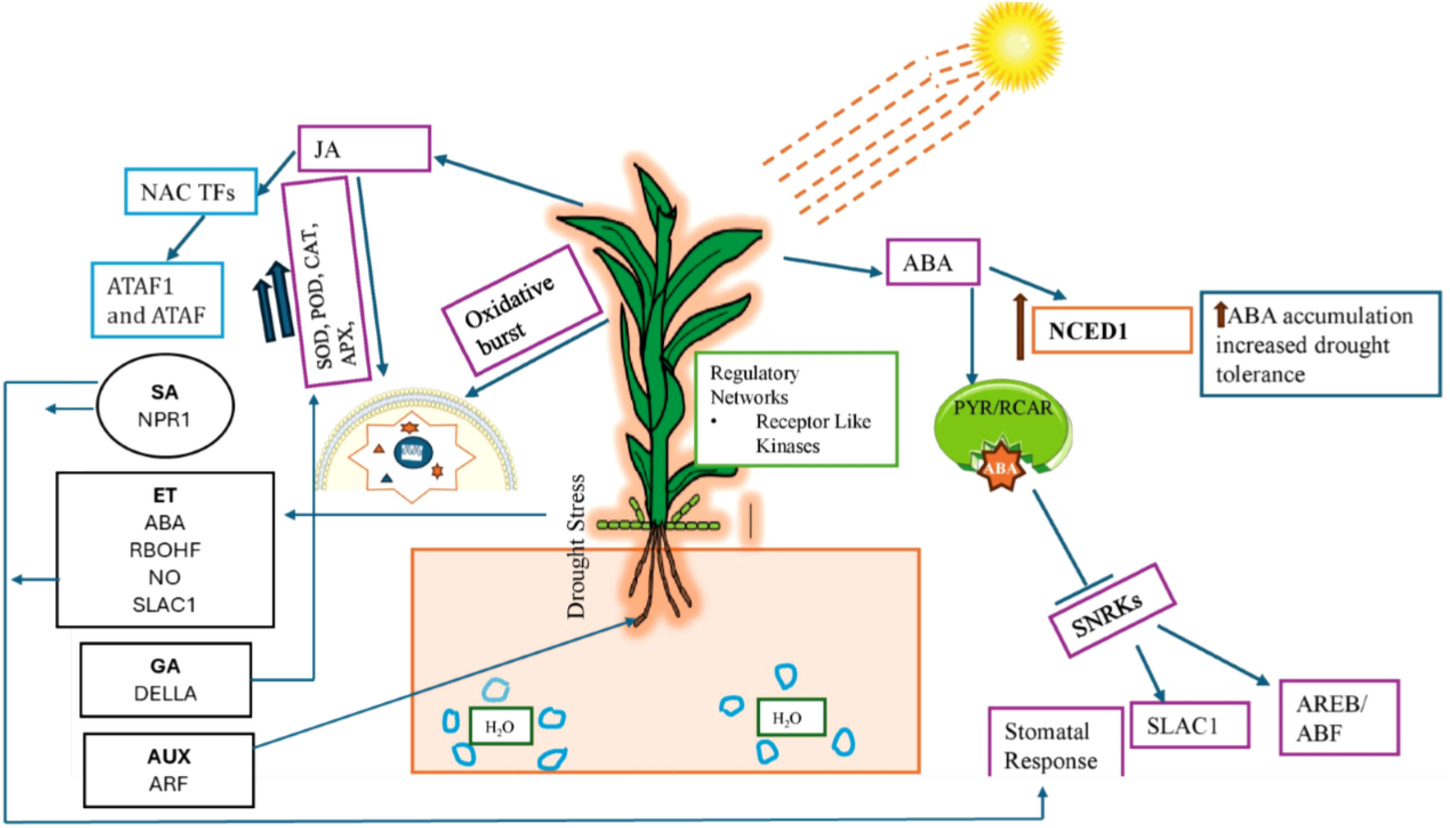
A schematic diagram shows the involvement of different hormones during drought stress in plants. ABA is the major hormone that regulates different responses in plants during drought stress such stomatal regulation by activating channels, transcription factors, receptors and signaling molecules. The role of other hormones such as SA, ET, AUX, CK and GA which regulates different responses such root system architecture, ROS detoxification and stomatal functioning is also highlighted in the figure.

In addition to ABA and JA, other hormones like SA, ET, auxin, BR, CK, and GA also play key roles in plant drought signaling. However, the specific role of each hormone at the molecular level remains unknown. Some reports have shown the positive and negative role of these hormones during drought stress in different crops. For example, exogenous application of SA improves plant biochemical and physiological adaptive traits during drought stress (Safari et al., 2021). Previous studies have shown that SA-deficient Arabidopsis mutant lines were more prone to drought stress and show more drought-induced detrimental symptoms than wild plants (He et al., 2014). Previous studies have shown that SA induces stomatal closure, inhibiting water loss and decreases CO_2_ assimilation during drought stress (Lee, 1998; Loutfy et al., 2012). SA mediated drought responses are driven by ROS activated ABA induced stomatal closure pathway, activating calcium channels and inducing calcium influx (Saito and Uozumi, 2019). However, SA can also regulate stomatal closure by enhancing ET synthesis via induction of *ACS2*, *ACS6*, and *ACS11* genes (Wang et al., 2020). These reports further showed that there is hormonal interplay between SA, ABA and ET that regulate plant responses during drought stress. ET regulates several plant growth and developmental processes, such as germination, fruit ripening, root-hair commencement, nodulation, leaf and flower senescence, abscission, and responses to a wide range of stressors (Fatma et al., 2022). However, its role in drought signaling is not fully understood. Some reports have shown that drought induces ET accumulation in plants which could control different growth and adaptive responses. For instance, ET accumulation during water stress leads to leaf abscission which reduces water loss (Mcmichale et al., 1972). Previous study has reported that ET mediated drought responses is primary regulated by hydrogen peroxide accumulation via RBOHF. The higher accumulation of ET in guard cells also contributes to the cleavage and nuclear localization of EIN2 through the activation of MKK1/3–MPK3/6 signaling cascade leading to stomal closure by modulating NO synthesis and SLAC1 channel activation (Zhang et al., 2021).In contrast, some studies have reported that ET can inhibit the ABA-induced stomatal closure or to reduce stomatal sensitivity during drought stress (Hassan et al., 2024). Therefore, it is important to decode how ET influences ABA signaling during drought stress and implications on growth and adaptive responses warrants future investigation. On the other hand, auxin has a positive influence on ABA signaling during drought stress. For example, exogenous application of auxin (IAA, indole-3-acetic acid) raised the ABA levels in *Trifolium repens* L. plants during drought stress which promotes drought tolerance by reducing water loss when compared to control plants (Zhang et al., 2023). Previous studies have found that auxin modulates the expression of key drought responsive genes such as *RAB18*, *DREB2A*, and *DREB2B*, *RD22*, *RD29A via ARF* induction (Shi et al., 2014; Zhang et al., 2020). During drought stress, ABA and auxin can play a key role in development and functioning by directing root growth towards water sources, to cope with water scarcity. However, there remains many knowledge gaps on how they regulate root system architecture at molecular levels therefore warrants future investigation. In plants BRs control different growth and other cellular processes such as cell elongation, seed germination, root architecture, pollen fertility, stomatal patterning, vascular development, and flowering. BR is known to affect drought tolerance in plants in both positive and negative ways. For instance, BR treated exogenously had increased drought tolerance, but BR mutants also exhibited drought tolerance (Ye et al., 2017). The decline in GA levels and increase of DELLA has been linked with enhanced drought resistance because low GA levels were shown to trigger the activation of various stress-related genes, accumulation of osmolytes and ROS detoxification (Tuna et al., 2008; Omena Garcia et al., 2019). Like other hormones, cytokinins also play a key role in drought stress resilience by delaying leaf senescence and sustaining photosynthesis (Rivero et al., 2007). In contrast, some studies have shown that cytokinin negatively regulates drought responses for instance, overexpressing cytokinin-deficient CKX- Arabidopsis plants showed increased resistance and survival during drought stress (Nishiyama et al., 2011). These studies showed that cytokinin can have both positive and negative impacts on plants during drought stress. However, how these responses occur at molecular level remains largely unknown. Hence, the coordinated control of plant hormone synthesis and synergistic and/or antagonistic interactions during drought stress provides evidence that phytohormone crosstalk is critical for balancing growth and adaptive responses during drought stress **Figure 3**.

## Drought stress memory in plants to defend future stress

Plants have an elegant sensory system to detect environmental cues and can memorize past stress events to protect themselves from future stress through a process called stress memory (Li and Liu, 2016). In a similar vein, plants can improve their resilience to subsequent drought events by employing drought stress memory, which entails many cellular modifications at the physiological, proteome, transcriptional, and epigenetic levels (Li and Liu, 2016). Drought stress memory has been observed in different crops such as *A. thaliana* (Van Dooren et al., 2020), *Brassica napus* L (Hatzig et al., 2018), wheat (Liu et al., 2021), rice (Zheng et al., 2013), to protect themselves from future drought stress. Plants that have had many drought stress exposures are better equipped to adapt to new stresses by changing their gene expression patterns more quickly than plants that have not experienced drought stress before (Li and Liu, 2016). These investigations imply that prior exposure to drought stress produced certain stress imprints that were preserved to promote recovery during a subsequent stressor. Although drought stress memory is seen from an evolutionary standpoint as a helpful strategy that could help a plant become more acclimated to withstand future stress however, some studies have linked it to adverse outcomes like stunted growth and development and decreased yield (Crisp et al., 2016; Wijewardana et al., 2019). Therefore, it will be interesting to investigate the growth defense tradeoffs during drought stress memory which will further provide novel insights on how it regulated growth and adaptive responses.

A growing body of evidence suggests that stress memory may involve transcriptional, translational, or epigenetic mechanisms to sustain the stress response. For instance, drought-responsive memory genes and the consequent improvement in transcriptional response to recurrent drought stress have been linked to histone marks, a type of chromatin alteration. In response to drought stress, the coding areas of drought-responsive genes RD20, RD29A, and AtGOLS2 showed a definite enrichment of H3K4me3, which persisted even after gene deactivation through rehydration, as reported by Kim et al. (2012). As a result, H3K4me3 is considered an epigenetic marker for drought stress memory in plants. Following a drought, selfed descendants of drought-stressed plants displayed higher levels of DNA demethylation in the ornithine-δ-aminotransferase (δ-OAT) and pyrroline-5-carboxylate synthetase (*P5CS*) genes compared to control treatments which further support the role of DNA demethylation in the development of stress memory in plants (Li and Liu, 2016). In plants, regulatory RNA like miRNAs and LncRNAs are also linked with drought stress memory because they were induced by drought stress and could be involved in drought tolerance (Yang et al., 2022). Also, the activation of transcriptional factors has been linked with drought stress memory response in plants. For instance, ABF TF transcript and protein levels revealed that ABF3 and ABF4 had transcriptional memory behavior despite only slightly elevated protein levels in response to recurrent drought stress (Virlouvet et al., 2014). Owing to the complexity of drought stress memory response more studies are required to identify key players that are involved in drought stress memory and growth defense tradeoffs. This will necessitate the use of multiomics tools and gene editing to decode the complexity of the drought stress memory process in plants.

## Multi-omics approaches to understand the molecular mechanism of drought stress tolerance in plants

Among environmental stressors drought stress severely affects plant growth and productivity. Various traditional and modern breeding approaches (molecular and genetic engineering) contributed significantly to understand the intricacy of the drought stress response (Hu and Xiong, 2014). A vast amount of data has been produced by recent advancements in omics methods, which are utilized to classify novel chemical and genetic factors controlling various physiological processes under stress. A single omics method may not always be particularly useful in determining the complexity of stress reactions; therefore, integration of omics tools is required to comprehend their molecular of complexity plant stress responses (Mir et al., 2022; Zargar et al., 2016). To tackle the challenges of omics data, a multitude of databases and tools are available for basic data analysis and interpretation. However, collaborative techniques are still necessary to evaluate physiological and biochemical changes generated by stressors. The integration of several omics techniques is essential to comprehending the biology of plant systems, which may be useful for stress tolerance engineering. Transcriptomics, metabolomics, proteomics, miRNA omics, and pan-omics, are some of the technologies that significantly impact plant response at the molecular level. Here, we present several branches and omics techniques for investigating the molecular and genomic aspects of plant drought responses.

Transcriptomics primarily aids in the identification of RNA or gene transcripts linked to a plant’s phenotypic expression under various environmental circumstances (Kwasniewski et al., 2016). A transcriptome investigation, such as RNA sequencing (RNAseq) and digital gene expression (DGE) are commonly used to decode transcriptional reprogramming in plants during drought stress which has led to the identification of numerous stress-tolerant genes. For instance, drought-tolerant sorghum line (SC56) showed induction of antioxidant genes (*SOD1, SOD2, VTC1, MDAR1, MSRB2, ABC1K1*), regulatory factors (*CIPK1* and *CRK7*) and repressors of senescence (*SAUL1)* (Azzouz-Olden et al., 2020). Meng et al. (2018), characterized the transcriptome of *Marsdenia tenacissima* in response to drought stress and discovered a variety of differentially expressed genes, such as *bZIP, bHLH, ERF, MYB, MYB*-related, and NAC families that are crucial for drought signaling and stress response. A comparative RNA seq study was carried out in two drought-tolerant wheat cultivars which reveals the identification of several DEGs related to oxidoreductase, heat shock protein, dehydrin, late embryogenesis abundant protein, sugar biosynthesis, and flavonoid biosynthesis, ABA linked TFs which plays a key role in drought resistance (Chu et al., 2021). On the other hand, following drought stress, rice RNA profiling revealed a variety of DEGs, including chlorophyll A-B binding protein, oxidoreductase GTPase activating protein, dehydrin, trehalose-6-phosphate synthase, and MYB transcription factor, which may be involved in drought adaptive response (Park and Jeong, 2023). Further, we have shown the list of RNA seq studies in different plants after drought stress in **Table 1**.

**Table 1:** Multi-omics approaches used to study the drought tolerance mechanism in different crops.

Crop	Cultivar used	Tissue Used	Single/Integrated omics analysis	Target gene/pathway identified	References
*Marsdenia tenacissima* (Rajmahal Hemp)	Yunnan	Roots, stems, and leaves	Transcriptome	*bZIP, bHLH, ERF, C2H2, MYB*, *MYB*-related, and *NAC* families	Meng et al., 2018
*Triticum aestivum* (Wheat)	TAM 111’ and ‘TAM 112	Leaves	Transcriptome	heat shock protein, oxidoreductase, late embryogenesis abundant protein and dehydrin, ABA-induced signal pathway	Chu et al., 2021
*Cicer arietinum* L. (Chickpea)	ICC 4958 (drought-tolerant) ICC 1882 (drought-sensitive)	Root	Transcriptome, proteome and metabolome	Genes, proteins, and metabolites involved in phosphatidylinositol signaling, glutathione metabolism and glycolysis/gluconeogenesis pathways	Singh et al., 2023; Kudapa et al., 2023
	ICC 4958, JG 11, and JG 11+ (drought-tolerant), and ICC 1882 (drought-sensitive)	Root	Transcriptome, proteome, and metabolome	Proteins encoding isoflavone 4’-O-methyltransferase, UDP-d-glucose/UDP-d-galactose 4-epimerase, and delta-1-pyrroline-5-carboxylate synthetase. Metabolites (fructose, galactose, glucose, myoinositol, galactinol, and raffinose)	
*Hordeum vulgare* (Barley)	SL-insensitive mutant hvd14 (dwarf14)	Leaf	transcriptomics, proteomics, phytohormonomics	Abscisic acid-responsive genes/proteins, lower jasmonic acid content, higher reactive oxygen species content, and lower wax biosynthetic and deposition	Daszkowska-Golec et al., 2023
*Zea mays* (Maize)	C7-2t	Leaf	Transcriptome, proteome, and metabolome	dehydrin, aquaporin, and chaperones to cope with osmotic stress, ABA, gibberellic acid galactinol-sucrose galactosyltransferase	Niu et al., 2024
*Citrullus lanatus* (Water melon)	black jade	Leaf	Transcriptome and metabolome	bHLH, MYB, HSP,GRF, ABA pathway, SnRK2-4 phenylpropanoids; polyketides, lignans, neolignans, carbohydrate, fatty acid and terpene glycoside	Chen et al., 2024
*Oryza sativa* (Rice)	Drought-sensitive (IR64) and a drought-tolerant (Nagina 22)	Seedling	Transcriptome, proteome, and metabolome	Gene/proteins responsible for L-phenylalanine biosynthesis Metabolites involved in aromatic amino acids and soluble sugars	Huang et al., 2014; Anupama et al., 2019; Abdirad et al., 2022
	PB6 (drought-tolerant indica rice variety), Moroberakan (drought-tolerant japonica rice variety) and Way Rarem (drought-sensitive indica rice variety)	Root	Transcriptome, proteome	Expression of Med37c and RSOsPR10 genes in transcriptomic profiling Role of chitinases from proteome analysis	
	IR64 (drought susceptible) and Azucena (drought tolerant)	Root tip meristem	Proteome	Proteins involved in root elongation viz., expansins and peroxidases were identified	
	H471 (drought tolerant) and HHZ (drought-sensitive)	Leaf	Transcriptome	Higher basal expression of oxidoreductase and lyase activities in tolerant plant	
	Rice	Root zone	Multi-omics	TFs involved in nitrogen metabolism, lipid metabolism, ABA signaling, ethylene signaling, and stress regulation were identified	
*Triticum aestivum* (Wheat)	Kukri (intolerant) Excalibur and RAC875 (tolerant)	Leaf	Metabolome	Amino acids viz., proline, tryptophan, leucine, isoleucine, and valine were increased	Bowne et al., 2012
Sugar maple (Acer saccharum Marshall)	Sugar maple	Saplings	Transcriptome	Dominant TFs identified: NAC, HSF, ZFPs, GRFs, and ERF Stress responsive genes: peroxidases, membrane transporters, kinases, and protein detoxifiers	Mulozi et al., 2023
Pinus halepensis Miller (Aleppo pine)	Aleppo pine	Whole plant	Transcriptome	Up-regulated genes: chlorophyll degradation, ROS-scavenging through AsA-independent thiol-mediated pathways, abscisic acid response and accumulation of heat shock proteins, thaumatin and exordium Downregulated genes: photosynthesis, ROS, fatty acid and cell wall biosynthesis, stomatal activity, and the biosynthesis of flavonoids and terpenoids	Fox et al., 2018
(*V. vinifera* cv. ‘Summer Black’) Grapevine		Leaf sample from 2 year old plant	Transcriptome	Induction of hormones: ABA, GA, BR, and IAA Upregulation of chlorophyll degradation and photosynthesis related genes	Haider et al., 2017
European beech (*Fagus sylvatica* L.)	European beech	Saplings	Transcriptome	Lipid- and homeostasis-related processes were upregulated whereas oxidative stress response genes were downregulated	Müller et al., 2017
Populus cultivars (*P. tremula × P. alba, P. nigra, P. simonii, P. trichocarpa*, and *P. tomentosa*)	10 *P. tomentosa* accessions	Leaf	Transcriptome	Candidate genes (e.g., PtoeIF-2B, PtoABF3, PtoLHCA4, and PtoPSB33) were identified	Song et al., 2022
C3 [*Oryza sativa* (rice)] and C4 [*Zea mays* (maize)] plant	Rice and maize plants	Whole plant	Transcriptome Meta-analysis	MAPK signaling pathway gets activated in both the plants RAB16B and RAB21 genes were upregulated MEI-like and PEAMT2 genes were downregulated	Tahmasebi and Niazi, 2021
Zea Maize (Maize)	Maize plant	Xylem sap	Metabolomic and proteomic	Changes in ABA, cytokinin hormones High romatic cytokinin 6-benzylaminopurine (BAP) Phenylpropanoid compounds changed (low lignin biosynthesis)	Alvarez et al., 2008
Alfalfa (*Medicago sativa* L.)	Zhongmu No. 1” (ZM) cultivar	Seed germination	Transcriptome	NCED, PYR/PYL, and PP2C may contribute to drought tolerance	Xu et al., 2024
*Astragalus mongholicus* (Mesoxerophyte) and *A. membranaceus* (Xerophyte)	Two Astragalus species (Tolerant and sensitive)	Seedlings	Transcriptome and metabolome	Upregulation of DREB gene expression and higher antioxidant efficacies in tolerant plant.	Liu et al., 2024
*Ipomoea batatas* *(Sweet potato)*	Drought tolerant and early maturing sweet potato variety	leaves	Transcriptome	*MYB, NAC (NAM, ATAF1/2, and CUC2), bZIP, ERF*	Arisha et al., 2020
*Z. mays*	A188, W22, and X178	leaves	Transcriptome	ABA biosynthesis, ABA co-receptors, *bzip4, bzip49, bzip68, bizp75, abf3*	Liu et al., 2021

Over the last ten years, an enormous amount of RNA sequencing data has been produced in various crop systems following drought stress. This data has yielded new insights into the intricacy of the transcriptional reprogramming drought signaling network, as well as the identification of many genes that are drought resilient and can be utilized to develop future drought-resistant plants. The resultant product of gene and protein activity, known as metabolites (primary or secondary), determines the impact on physiological activity and other aspects of living phenotype (Fiehn et al., 2000; Rinschen et al., 2019). Primary metabolites are vital for plant growth and have a broad function in physiological activity, while secondary metabolites are crucial for defense responses in the face of a variety of abiotic challenges (Mashabela et al., 2022; Verslues et al., 2023). Drought has been shown to promote the accumulation of several secondary metabolites, including complex phenols, terpenes, and alkaloids. Drought stress, for instance, increases the phenolic content of rice, barley, garden sage, and hypericum. Similarly, during drought stress, garden sage and barley both exhibit increases in the number of monoterpenes or terpenoids (Quan et al., 2016; Piasecka et al., 2017; Radwan et al., 2017). Likewise, under drought conditions, trehalose, a non-reducing disaccharide, helps to preserve membrane integrity and stabilize macromolecules. Numerous other primary metabolites, like organic acids, are significant for various forms of abiotic stress. Malic acid, for instance, confers drought resistance in a variety of plant species, including cotton, spare grasses, and tropical grasses (Sharma and Dubey, 2019). In potato genotypes, overexpression of galacturonic acid reductase contributes to increased ascorbic acid concentration and water stress resistance (Chaturvedi et al., 2022). A recent comparative metabolomics study in contrasting genotypes under drought stress reveals the accumulation of differentially abundant metabolites (DAMs) in drought-tolerant and susceptible rice genotypes, such as amino acid biosynthesis, purine metabolism, fatty acid biosynthesis, TCA cycle, and starch and sucrose metabolism (Hamzelou et al., 2022). Similarly, following drought stress, a metabolomics investigation on wheat revealed that the primary metabolites altered in abundance due to water scarcity were amino acids, organic acids, and sugars (Michaletti et al., 2018). Numerous metabolomics investigations conducted on plants under drought stress have produced a snapshot of the differently accumulated compounds, which are summarized in **Table 1**.

Proteomics is another omics-based approach to investigate how drought stress can affect the proteome in plants. To better understand the molecular mechanisms underlying plant species’ resistance to drought, the proteomic method has been applied in the past to a variety of plant species, including *B. napus* (Koh et al., 2015), rice (Wan and Liu, 2008), soybeans (Das et al., 2016). These studies have reported the expression of differentially expressed proteins involving diverse plant growth and adaptive traits. Deng et al. (2018), studied comparative proteome analysis in wheat plants after drought stress and found differentially accumulated proteins such as (DAP) like ADP glucose pyrophosphorylase (AGPase), rubisco large subunit (RBSCL), oxalate oxidase 2 (OxO_2_) and chaperonin 60 subunit alpha (CPN-60 alpha) which might be involved in drought resistance. In rice plants, proteomic profiling after drought stress shows the abundance of differentially accumulated proteins such as LTP1, DHAR1, HSP 18.6, KAT, TIM18, RNS3, GRXC6, and ADF3 which might have a role in drought adaptive response (Hamzelou et al., 2020). Further, we have shown the proteomic studies that highlight the expression of differentially accumulated protein in plants under drought stress in **Table 1**.

Ionomics is another important omics tool to examine the compositions of metals, non-metals, and metalloids in plants. Ions are crucial for plant development and stress tolerance, and changes in them can be harmful to plants (Ali et al., 2021). Drought stress has a severe impact on mineral nutrition in plants. For example, drought stress affects the absorption of Fe, Zn, Mn, and Mo in *B. napus* which hurts plant survival (D’Oria et al., 2022). On the other hand, the exogenous application of ions in plants improves drought tolerance. For instance, sulphur-based fertilizers increase the rate of photosynthesis, stomatal conductance, transpiration rate, and antioxidant generation in maize plants, hence improving drought tolerance (Usmani et al., 2020), Potassium is essential for plants to be able to withstand drought. For instance, stomatal closure in sunflowers cultivated in drought-prone environments is anticipated by the guard cells through the ethylene pathway, resulting in a higher photosynthetic rate than in plants grown in low-K circumstances (Islam et al., 2024). Previous research has shown that increasing photosynthetic activity by N supplementation and activating an antioxidative defense system in wheat can reduce the effects of drought stress (Abid et al., 2016). Salicylic acid, on the other hand, increases the uptake of macro and micronutrients, such as P, Fe, Mn, and Zn, which in turn improves plant water relations, stomatal regulation, cell membrane stability, osmolyte accumulation, water use efficiency, and photosynthesis, thereby mitigating the effects of drought stress (Brito et al., 2019). The ability of canola genotypes with higher S consumption efficiency (SUE) to tolerate drought stress was demonstrated by Lee et al. (2016). In a similar way, *Vigna radiata* was also shown to be resistant to drought with enhanced K buildup (Islam et al., 2024). These studies further highlight the importance of plant ions in drought tolerance. In addition to shedding light on the role of the plant ionome during drought stress, future research on ionomics and its integration with other omics data will open new avenues for determining the genes and regulatory pathways involved in mineral uptake, transportation, and molecular mechanisms under both normal and drought stress conditions.

Drought resistance (DR), a complex quantitative trait involves molecular, morphological and physiological responses (Yang et al., 2020). One of the main goals of modern plant biology is to have a thorough understanding of the link between genotype and phenotype, which requires reliable identification and description of crop phenotypic traits for the crop improvement. Various phenotyping tools like red-green-blue (RGB), hyperspectral imaging, thermal infrared (IR) and chlorophyll fluorescence are most widely used for examining drought related traits (Mulugeta Aneley et al., 2023). For instance, numerous traits including leaf area, plant height, water status, biomass, photosynthetic efficiency, transpiration, and pigment content, can be determined using these phenotyping tools (Mulugeta Aneley et al., 2023). Previously, LIDAR-based phenotyping was used to evaluate drought induced morphological changes in drought tolerant and sensitive potato plants. Based on their findings, drought-tolerant genotypes under drought stress produced shorter shoots, quicker shoot growth, longer leaf area growth and larger projected leaf area than sensitive plants. Moreover, under drought stress, tolerant plants kept their lower leaf angle at daybreak, mimicking those of unstressed plants (Findurová et al., 2023). Similarly, different phenotypic tools such as thermal infrared, chlorophyll fluorescence and red-green-blue (RGB) were used to monitor morphological and physiological traits in different drought barley genotypes which shows that they differ in their stomatal conductance, wilting, leaf area (Fehér-Juhász et al., 2014). These investigations not only enable testing of the yield performance of novel barley genotypes under drought stress, but are also essential for choosing the genetic resources for the ensuing breeding process. In transgenic wheat plants, phenotyping tools were used to study drought tolerant traits such as plant length, water content, and other physiological traits. To overcome the obstacles in genomic and phenomic research, high-throughput phenotyping (HTP) is becoming more popular as a phenomic technique because it can analyze vast amounts of phenotypic data accurately a precisely. HTP was used to study different physiological and molecular traits in six chickpea genotypes. They found that during drought stress, there were variations in the following attributes: dry weight and projected area, plant height, stomatal conductance, chlorophyll fluorescence, caliper length, convex hull area, photosynthetic rate, and IR thermometer temperature traits within 6 chickpea genotypes and these traits can be further used in breeding programs to enhance drought tolerance (Pappula-Reddy et al., 2024). Similarly, HTP was used to examine drought tolerant traits in different tomato genotypes which can be further used for it drought improvement (Genangeli et al., 2023). Duan et al. (2018) use HTP to study different morphological and physiological traits in drought tolerant and sensitive rice genotypes which provide novel insights on their drought response. In maize plants, high-throughput multiple optical phenotyping was used to decipher the genetic architecture of 368 maize genotypes for drought tolerance (Wu et al., 2021). Based on the above studies, HTP tools can distinguish between resistant and susceptible drought plants which can save time for breeding cycles. For drought experiments, high-throughput phenotyping is becoming more and more popular as a means of overcoming the obstacles encountered in genomic and phenomic research. Large-scale phenotypic data analysis may be done accurately and non-destructively by researchers using HTP during drought stress in different crops systems.

## Integrative omics approaches for drought stress tolerance

**Figure 4:**
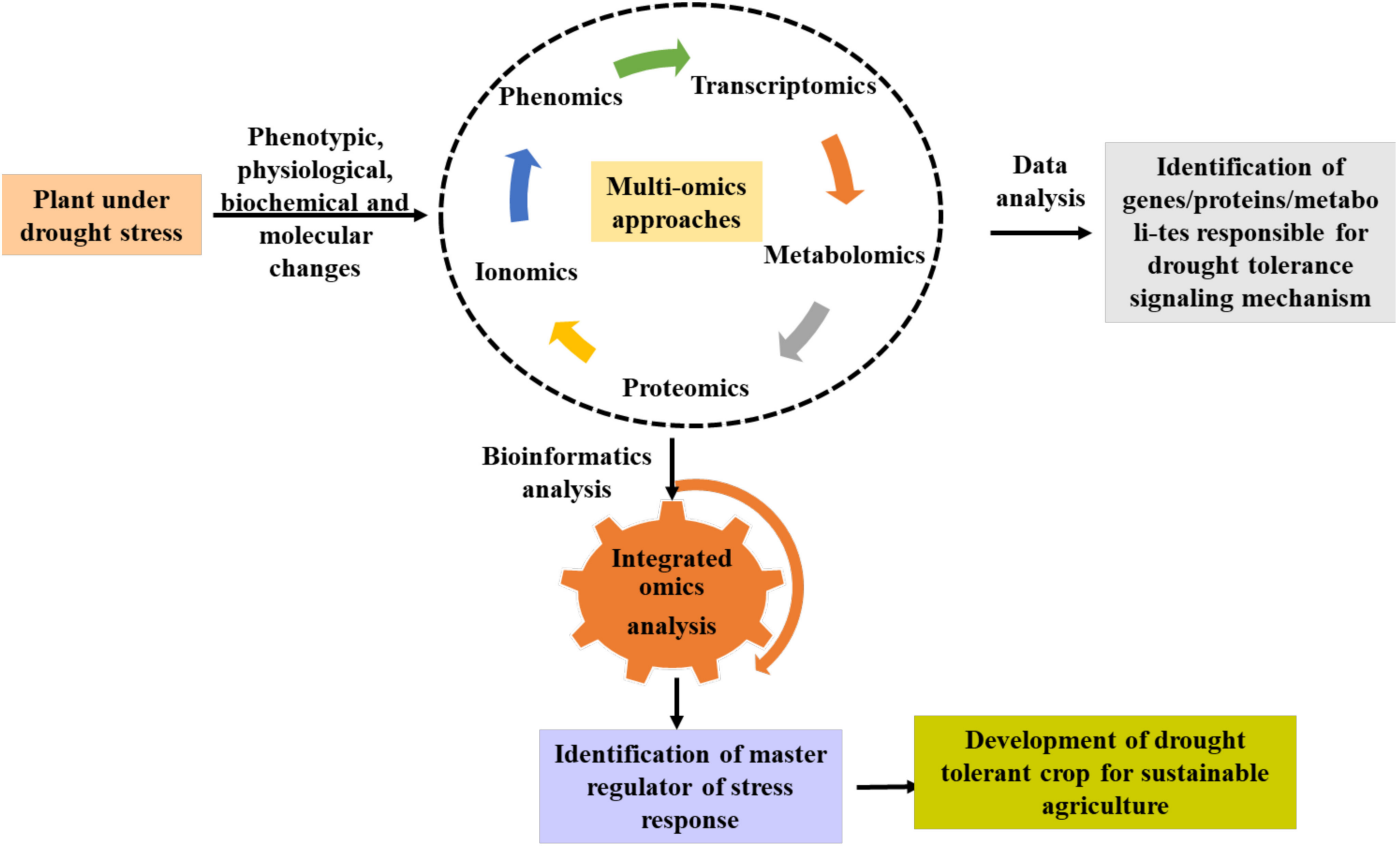
A schematic diagram shows the role of omics tools for deciphering the molecular complexity of drought response in plants. Integrative omics approach can aid in the identification of potential genes, metabolites or proteins that can be used for developing future drought tolerant crops using gene editing.

The integration of several omics tools has been instrumental in unwiring the molecular complexity of complex traits associated with plant stress and growth responses. A few integrative omics studies have been used in different plants to decode stress responses. For instance, a combined transcriptomic, proteomics, and metabolomics approaches were used to study the biochemical changes in *Quercus ilex* during drought stress which resulted in the identification of potential drought response candidates (*DEBR2A, WRK65*, ClpB proteases, FTsH6 protease, APX2, and glutathione S-transferase and proline) that can be used for improving drought resilience (Guerrero-Sánchez et al., 2022). Utilizing transcriptome, proteomics, and metabolomics provided novel insights in oil palm for salinity and drought response (Bittencourt et al., 2022). By combining data from rootomics, it was possible to identify several significant candidate genes that underlie drought-responsive “QTL-hotspots” and to understand the drought response in chickpeas (Kudapa et al., 2023). Singh et al. (2023) also identified co-expressed genes, proteins, and metabolites implicated in phosphatidylinositol signaling, glutathione metabolism, and glycolysis/gluconeogenesis pathways by an integrated multi-omics analysis of transcriptome, proteome, and metabolome data, particularly in the drought tolerant chickpea genotype. On the other hand, a multiomics approach was used to decipher transcriptional, and metabolomic reprogramming in water melon during drought stress. This revealed differentially expressed genes (bHLH, MYB) and metabolites (phenylpropanoids; polyketides, lignans, neolignans, carbohydrate, fatty acid, and terpene glycoside) that may be crucial for plant survival and growth under drought conditions (Chen et al., 2024). Trio omics methods using the transcriptome, proteome, and metabolome of maize plants allowed for the identification of several osmotic stress-resistance factors, including gibberellic acid, dehydrin, aquaporin, chaperones, ABA, and galactinol-sucrose galac-tosyltransferase (Niu et al., 2024). In barley, integrative omics studies led the identification of differentially expressed genes/proteins and metabolites such as abscisic acid-responsive genes/proteins, jasmonic acid content, higher ROS content, and lower wax biosynthetic and deposition (Daszkowska-Golec et al., 2023). These studies provide novel insights on drought induced molecular and metabolic changes in plants that can be used for crop improvement. A greater comprehension of the regulatory networks and molecular mechanisms controlling crop responses to abiotic stress can be achieved by integrating multi-omics data. This information can be used to identify prospective biomarkers or targets for future crop improvement, as well as to generate drought stress-tolerant crop types using focused breeding and genetic engineering (transgenic and genome editing) techniques **Figure 4**.

## Genome manipulation strategies for the development of drought-tolerant crops

Plants respond to drought stress by regulating a large array of molecular processes controlled by differential gene networks (Liu et al., 2023; Zhang et al., 2018). For instance, in maize drought stress induced upregulation of genes coding for transcription factors such as *bHLH*, *bZIP, ERF*, and *NAC* critical for growth and development (Liu et al., 2023; Zhang et al., 2018). Zhang et al. (2018), identified the *ZmNAC111* transcription factor which was up-regulated under drought stress in maize by using deep sequencing analysis. Additionally*, SiHDZ13* and *SiHDZ42* genes coding TFs are up-regulated under drought stress conditions in sesame (*Sesamum indicum* L.) (Wei et al., 2019). Similarly, *HD-ZIP* TFs and *DREB* genes belonging to AP2/ERF were found to be key players in controlling drought stress in several plant species (Sharif et al., 2021). These studies indicate the role of a specific set of TFs critically in alleviating drought stress in crop plants. In addition to TFs, several studies unravel the role of diverse classes of proteins viz., sucrose nonfermenting1 (SNF1)-related protein kinases (SnRKs), calcium-dependent protein kinases (CDPKs/CPKs) and mitogen-activated protein kinase (MAPK) cascades which controls, ABA- and NAC-mediated signaling pathways to enhance resilience against drought stress (Wang et al., 2019). For instance, the SlSnRK2.4 gene was overexpressed under drought stress to regulate the ABA signaling pathway, suggesting its pivotal role in alleviating role in circumventing drought stress in plants (Liu et al., 2021). For instance, reports suggest that proline contributes largely to tolerance against drought stress (Karthikeyan et al., 2011; Surekha et al., 2014). Perspectives to this, Kishor et al. (1995), developed a transgenic *Nicotiana tabacum* by transferring P5CS under the control of CaMV 35S promoter from *Vigna aconitifolia* leading to 10 fold increase in proline content and consequent tolerance to drought stress. Similarly, Zhu et al. (1998), developed a transgenic rice plant with elevated levels of proline by transferring the *P5CS* gene from *V. aconitifolia* to hence salt and drought tolerance. Zhang et al. (2015), developed transgenic soybeans overexpressing the StP5CS gene to tolerate salt stress and drought stress. Recently, Yang et al. (2023), developed a transgenic soybean plant ectopically expressing AtSINA2 gene product increasing grain yield, enhancing shoot growth and flowering, decreasing malondialdehyde (MDA) accumulation and preventing water loss under drought stress conditions. Similarly, under the control of the promoter of drought stress-induced gene *OsHAK1, OsSUT1* was overexpressed to promote transport sugars from source to sink to prevent water loss, prevent lipid peroxidation, enhance expression of stress-responsive and antioxidant genes to improve drought stress tolerance in rice plants (Chen et al., 2024). In conclusion, transgenic approaches play a central role in enhancing food production by transfer of desired genes from wild or cultivated relatives to produce drought-tolerant crop plants (Basit et al., 2024; Şimşek et al., 2024; Wang et al., 2024).

The last decade has witnessed the emergence of genome editing (GE) techniques as forefront techniques to precisely edit plant genomes for developing next-generation resilient crops (Rai et al., 2023; Bhat et al., 2021; Tariq et al., 2023; Yaqoob et al., 2023). Clustered regularly interspaced palindromic repeats (CRISPR)/Cas9 is reported to be the most efficient in manipulating plant genomes among a wide range of reported CRISPR systems (Brokowski and Adli, 2019). The CRISPR-Cas9 utilizes small guide RNA molecular and a protein part possessing endonuclease activity that precisely alters the genomic DNA strands to generate dsDNA breaks. These breaks are subsequently repaired by cellular repair mechanisms, resulting in novel variations in target genes (Bouzroud et al., 2020). Consequently, this GE is effectively employed to achieve tolerance against multiple types of abiotic stress factors viz., drought stress, salinity stress, heat and cold stress. Reports suggest that AREB1 protein inactivation in crop plants increased susceptibility to drought stress, whereas overexpression of this protein helped in understanding drought stress. Consequently, AREB1 was also reported to regulate the expression of a wide range of proteins linked to ABA biogenesis, ABA signaling, antioxidant signaling, and osmotic stress response (Roca Paixão et al., 2019). Perspectives to these reports, CRISPR-Cas9 combined with sgRNA and dead Cas9 has been employed to unravel the AREB1 activity by editing promoter regions in Arabidopsis (Roca Paixão et al., 2019). These manipulations led to the conclusion that AREB1 has a positive impact on alleviating plants against drought stress. In rice, CRISPR-Cas9 technology was operated to produce mutants of osmotic stress/ABA-activated protein kinase 2 (SAPK2) impairing the ABA signaling, these rice mutants were found considerably sensitive to drought and oxidative stress. Thus, these studies concluded that SAPK2 is a promising gene critical for raising drought-tolerant crop plants (Lou et al., 2017). Similarly, CRISPR/Cas9 was under the control of tissue-specific promoter AtEF1 led to the effective induction of mutations in OPEN STOMATA 2; OST2;PLASMA MEMBRANE PROTON ATPASE/H(+)-ATPASE 1 (*OST2/AHA1*) genes responsive to drought stress (Martignago et al., 2020). Moreover, CRISPR-Cas9 has been effectively employed to precisely target genes such as *SlEPSPS, SlARF4, SlcBF1*, *SlHyPRP1*, and *SlBZR1* to produce tomato plants resilient to abiotic stressors like drought, heat, cold, and salinity stressors (Chen et al., 2021; Tran et al., 2021; Yin et al., 2018; Li et al., 2018; Yang et al., 2022). On the hand, in rice plants silencing of negative regulators of drought tolerance like *Oryza sativa* stress related ring finger protein 1 (OsSRFP1), drought induced SINA protein 1 (OsDIS1), and drought and salt tolerant protein 1 (OsDST) confers drought tolerance (Ning et al., 2011; Huang et al., 2009; Fang et al., 2015; Wang et al., 2017; Ogata et al., 2020). Wang et al. (2017) used CRISPR/Cas9-based approach to study the role of MAPK3 in drought signaling in tomato plants. According to their findings, plants that have MAPKs silenced are more susceptible to drought stress since these proteins control several drought responses. In rice, silencing of ENHANCED RESPONSES TO ABA1 (ERA1) protein boosts drought tolerance as compared wild plants which further shows its key role in drought response regulation (Ogata et al., 2020). Previous study has shown that knockout of auxin response factor (ARF) in tomato plants confers drought tolerance (Chen et al., 2021). Osakabi et al., 2016, edited *OST2* gene in Arabidopsis which leads drought tolerance by modifying the stomatal functional traits. on the other hand, many researchers have targeted DREB transcriptional factors using genome editing approaches which has been instrumental in understanding their role in drought response in plants (Arroyo-Herrera et al., 2016; Kim et al., 2018). Li et al. (2019) used CRISPR/Cas9 system to target *NPR1* gene in tomato plants with an aim to study its role in drought tolerance. However, based on their findings it was found that tomato plants become more vulnerable to drought due to wider stomatal aperture, low antioxidant enzymes and greater electrolytic leakage which further highlights the role of SA pathway in drought tolerance. Recently, phylloplanin-like gene in maize was edited by CRISPR-Cas9- which improves drought tolerance than wild plants (Wang et al., 2025). In *Glycine max* CRISPR-Cas9 editing of *GmHdz4* transcription factor confers drought tolerance (Zhong et al., 2022). All these reports demonstrate the versatility of CRISPR-Cas9-based GEs play a critical role in improving crop plants to develop resilience against a wide range of abiotic stressors, especially against drought stress. The identification of target genes both negative and positive regulators, using gene editing tools are important for crop breeding programs.

## Conclusion and future directions

Drought is one of the major environmental stressors that significantly impact plant growth and development, especially in regions with low water availability or rainfall. Improving drought stress tolerance in crops is one of the major goals in plant stress biology and crop breeding programs. However, owing to the molecular complexity of drought responses in plants, researchers are facing many obstacles to finding the elite traits which could provide long durable resistance to drought stress. Also, there remains many knowledge gaps in understanding how plants perceive drought signals and triggers signal transduction which regulates adaptive and stress responses. So far, many drought induced channels, receptors, secondary signaling messengers, hormones, and gene networks have been identified in different models and crop plants. However, their usage for developing long term drought resileint crops is still challenging. Therefore, more studies are required to unwire the complexity of drought responses in plants. Plant growth and stress response during drought stress are intricate biological processes that require system-level analysis utilizing physiological and genetic methods to identify major signaling players involved which can be used for developing drought tolerant crops. In this regard, utilizing omics as well as pan omics-based tools can offer fresh perspectives on the molecular and biochemical alterations that plants experience during drought stress. These insights can then be utilized to identify specific genes or networks that can be utilized to create drought-tolerant plants using genome editing techniques. On the other hand, how plants develop drought resistance memory warrants future investigation to unwire the molecular intricacy of drought priming in plants. It is well documented that several chemical signals travel from roots to shoots once plants first detect drought stress in their roots which modulates an array of growth and adaptive responses. Some players like ABA, peptides, ROS and calcium are known to play an important role in long distance signaling during drought stress. However, their molecular interplay is not fully understood therefore warrants future investigation. Finally, how drought affects microbiome structure and changes root exudates chemistry requires future investigation. The main objective of this review was to provide a systemic overview of the existing knowledge about how drought induced signaling cascades leads to robust responses against water scarcity in plants. From an evolutionary perspective, plants can withstand drought stress by balancing their growth and stress responses which are however very complex and interconnected by diverse signaling molecules and may vary from plant species. Therefore, unravelling this molecular intricacy –and identifying new adaptive traits can offer novels avenues for improving drought tolerance in plants using biotechnological or breeding programs.
